# Childhood’s chronic headache in children’s national hospital of Albert Royer in Dakar

**DOI:** 10.1186/1129-2377-14-S1-P33

**Published:** 2013-02-21

**Authors:** LB Seck, NS Diagne, AD Sow, M Ndiaye, L Gueye, D Sow, AG Diop, MM Ndaiye

**Affiliations:** 1 Fann Teaching Hospital, Senegal; 2 Albert Royer Children National Hospital, Senegal

## Introduction

Children's headaches are frequently primary. Their diagnosis is difficult because of anamnesis discomfort. Aetiologies are numerous, above all functional ones as migraine. As in adults, symptomatic headache has to be taken into account among these.

## Purpose

Our aim is to determine the clinical and aetiological features in a sample of children suffering from headache.

## Methods

This was a prospective study conducted at the Children National Hospital Albert Royer (CHNEAR) in Dakar. We included children aged 5-15 years, received in out-patient department for chronic headache. The marital status, personal and family past medical history, headache characteristics, physical examination data were searched and supplemented with additional tests according to the clinical context.

## Results

We collected 43 children. The sex ratio was 1.05 in favour of girls. The mean age was 10.68. Fifty-five point eight percent of them had familial past medical history of chronic headache in at least one of the two parents. It was localized headache in 76.19% of the cases and diffused in 21.42% of them. The most frequent localized headaches were frontal or fronto-orbital (35.71%), temporal (19.4%), hemicranial (16.66%). The main triggering factors were noisy atmosphere (60.40%), light (37.20%), fatigue (35%), heat (28%), and nervousness (25.50%). The brain CT- scan was performed in 25.50% of children and had returned normal except in one case. The EEG performed in 14 patients did not find any abnormality. Migraine was present in 58.13% of cases, non specific headaches in 41.80% of cases, hypertension induced headache in one case (2.3%). Associated pathologies were psychomotor developmental delay (11%) and seizures (11.62%).

## Conclusion

Children chronic headaches are frequently primary ones, above all migraine. Sensory and psychic factors are the most frequent among triggering factors. As in adults, atypical features must lead to cerebral lesion research.
